# *DICER1* and microRNA regulation in post-traumatic stress disorder with comorbid depression

**DOI:** 10.1038/ncomms10106

**Published:** 2015-12-03

**Authors:** Aliza P. Wingo, Lynn M. Almli, Jennifer J. Stevens, Torsten Klengel, Monica Uddin, Yujing Li, Angela C. Bustamante, Adriana Lori, Nastassja Koen, Dan J. Stein, Alicia K. Smith, Allison E. Aiello, Karestan C. Koenen, Derek E. Wildman, Sandro Galea, Bekh Bradley, Elisabeth B. Binder, Peng Jin, Greg Gibson, Kerry J. Ressler

**Affiliations:** 1 Atlanta VA Medical Center, Atlanta, Georgia 30033, USA; 2 Department of Psychiatry, School of Medicine, Emory University, Atlanta, Georgia 30322, USA; 3 McLean Hospital, Harvard Medical School, Belmont, Massachusetts 02478, USA; 4 Department of Psychology, University of Illinois at Urbana-Champaign, Illinois 61820, USA; 5 Carl R. Woese Institute for Genomic Biology, Illinois 61820, USA; 6 Department of Human Genetics, School of Medicine, Emory University, Atlanta, Georgia 30322, USA; 7 Department of Psychiatry and MRC Unit on Anxiety and Stress Disorders, University of Cape Town, Cape Town, South Africa; 8 School of Global Public Health, University of North Carolina at Chapel Hill, Chapel Hill, North Carolina 27599, USA; 9 Harvard T.H. Chan School of Public Health, Boston, Massachusetts 02115, USA; 10 School of Public Health, Boston University, Boston, Massachusetts 02118, USA; 11 Center for Integrative Genomics, School of Biology, Georgia Institute of Technology, Atlanta, Georgia 30332, USA

## Abstract

*DICER1* is an enzyme that generates mature microRNAs (miRNAs), which regulate gene expression post-transcriptionally in brain and other tissues and is involved in synaptic maturation and plasticity. Here, through genome-wide differential gene expression survey of post-traumatic stress disorder (PTSD) with comorbid depression (PTSD&Dep), we find that blood *DICER1* expression is significantly reduced in cases versus controls, and replicate this in two independent cohorts. Our follow-up studies find that lower blood *DICER1* expression is significantly associated with increased amygdala activation to fearful stimuli, a neural correlate for PTSD. Additionally, a genetic variant in the 3′ un-translated region of *DICER1*, rs10144436, is significantly associated with *DICER1* expression and with PTSD&Dep, and the latter is replicated in an independent cohort. Furthermore, genome-wide differential expression survey of miRNAs in blood in PTSD&Dep reveals miRNAs to be significantly downregulated in cases versus controls. Together, our novel data suggest *DICER1* plays a role in molecular mechanisms of PTSD&Dep through the *DICER1* and the miRNA regulation pathway.

## Abstract (web summary)

DICER1 is required for the maturation of miRNAs which regulate expression of thousands of genes. Here the authors show significantly reduced levels of *DICER1* in individuals having post-traumatic stress disorder and comorbid depression suggestive of a role in the molecular mechanism of the condition.

## Graphical abstract

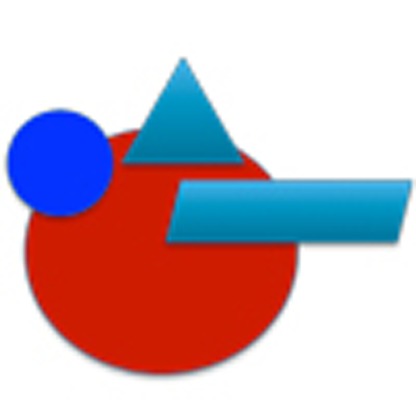

Exposure to traumatic life events and its sequelae, such as post-traumatic stress disorder (PTSD) and depression, are a significant source of suffering and impairment around the world and major contributors to the global burden of disease^1^,^2^. However, our understanding of biological mechanisms underlying PTSD and depression is still incomplete. Gene expression profiles reflect dynamic interplay between genes and the environment and thus can provide a window into molecular mechanisms underlying these stress-related disorders. Though gene expression is tissue specific, expression levels of many genes have moderate correlations between blood and other tissues, including post-mortem brain^3^,^4^. Moreover, stress-related transcriptomic changes in peripheral blood mononuclear cells were paralleled by stress-related gene expression changes in the relevant brain regions in mice^5^. Since it is difficult to obtain brain tissues in live human subjects, gene expression profiles in blood may provide a useful surrogate to offer information on biological mechanisms of neuropsychological disorders as well as their effects on physical health.

PTSD with comorbid depression (referred to as PTSD&Dep from here on) is highly prevalent among susceptible individuals following traumatic life experiences. For example, in the Grady Trauma Project (GTP) sample of 6863 at-risk inner city participants with high rates of trauma exposure, the prevalence of PTSD&Dep was 28.4%. In another instance, among the 29,486 post-9–11World Trade Center Health Registry enrolees, 4.8% had depression only, 5.1% had PTSD only and 10.1% had PTSD&Dep at 10 years after the September 11 disaster^6^. Nosologic distinctions among different psychiatric disorders so far have largely been based on descriptive studies and not on neural circuits or biological mechanisms. Hence, in this study, we aimed to increase our understanding of molecular mechanisms underlying PTSD&Dep, a relatively common sequel of traumatic life events.

To that end, we survey genome-wide differential gene expression profiles in blood in cases of PTSD&Dep and traumatized controls with no PTSD and no depression. Our hypothesis is that genes differentially expressed in PTSD&Dep inform biological pathways underlying this psychiatric phenotype. We find that expression of *DICER1* is significantly reduced in the cases relative to the controls, and this finding is replicated in two independent cohorts. This is quite intriguing given prior possible association of *DICER1* to depression-related phenotypes in humans and mice^7^,^8^. To follow-up on the *DICER1* finding, we perform expression quantitative trait locus (eQTL) analysis for *DICER1* and find an eQTL, rs10144436, located in *DICER1*'s three prime un-translated region (3′UTR), significantly associated with PTSD&Dep and replicate this finding in another cohort. Additionally, we find that lower blood *DICER1* expression is significantly associated with increased activation of the amygdala to threat stimuli, a neural correlate of PTSD, in a functional magnetic resonance imaging (fMRI) study. *DICER1* is notable as an enzyme that is central to the post-transcriptional regulation of gene expression as it cleaves precursor microRNAs (miRNAs) and double-stranded RNAs into mature miRNAs and short-interfering RNAs, both of which regulate expression of thousands of genes post-transcriptionally^9^. Given *DICER1* functions, we survey genome-wide differential miRNA expression profiles in blood in a smaller discovery sample of PTSD&Dep and find two miRNAs significantly reduced in abundance in the cases. Of these, miR-3130-5p is also significantly downregulated in PTSD&Dep cases versus controls in a replication sample, and one of its mRNA targets is upregulated in the PTSD&Dep cases. Together, our data show that *DICER1* and miRNAs are involved in molecular mechanisms of PTSD&Dep via the *DICER1* and the miRNA regulation pathway. This stress-related *DICER1* and miRNA regulation in blood is paralleled by recent findings of stress-related *DICER1* and miRNA regulation in brain in a mouse model^8^.

## Results

### Characteristics of the discovery cohort

There were 184 participants in the GTP discovery cohort (Table 1). Of these, 112 were cases of current PTSD&Dep and 72 were traumatized controls with no current PTSD and no current depression. Specifically, mean PTSD symptom scores among the cases and controls were 28.7 and 2.1, respectively, and mean depression scores among the cases and controls were 28.2 and 2.7, respectively (Table 1). The cases and controls had comparable age and proportions of men and women (Table 1). As expected, the cases were exposed to more traumatic life events than the controls (7.3 versus 3.8; Table 1). In a subset of these participants (*n*=145–150), we had information on current alcohol and drug use disorders, bipolar disorder and current psychotic disorder assessed by the Mini International Neuropsychiatric Interview (MINI)^10^ and Structure Clinical Interview for DSM-IV (SCID)^11^. More cases had substance use disorder, psychotic disorder and bipolar disorder than controls, but these differences did not reach statistical significance (Table 1). Lastly, the cases had slightly more percentage of African Americans than the controls (Table 1). We controlled for these psychiatric variables and ancestry in our differential expression analysis as detailed below.

### Genome-wide differential expression profiles in PTSD&Dep

**Figure 1:**
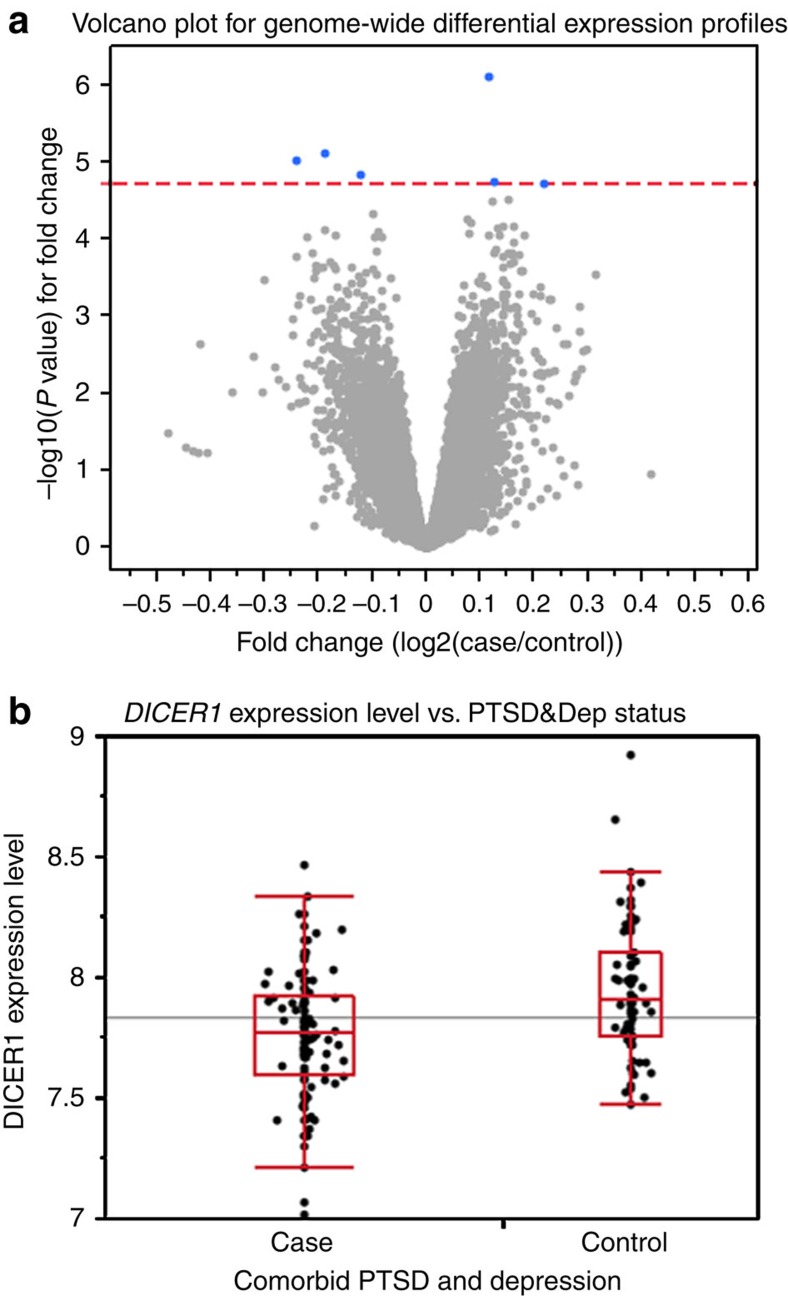
Genome-wide differential gene expression profiles in PTSD&Dep cases vs controls. (**a**) Volcano plot for genome-wide differential gene expression profiles between cases of PTSD&Dep and controls, adjusting for sex, age and population substructure. The red line represents *P* value at FDR of 0.05 (−log_10_p=4.729). The blue dots represent genes significantly differentially expressed between cases and controls. These genes are *MRPS23*, *DICER1*, *CHST15*, *ZXDC*, *PDCD5* and LOC649548. Cases had reduced *DICER1* expression compared with controls. (**b**) Box plot for *DICER1* expression level in cases of PTSD&Dep and controls. The horizontal line represents the mean *DICER1* level in the overall sample. These were derived from expression microarrays.

In our primary analysis, at a genome-wide false discovery rate (FDR) <0.05, we found six genes significantly differentially expressed between cases and controls after controlling simultaneously for sex, age and population substructure via genotypic principal components (Fig. 1). In order of the lowest to highest adjusted *P* value, these genes were *MRPS23* (mitochondrial ribosomal protein S23), *DICER1* (DICER1, ribonuclease type III), *CHST15* (carbohydrate *N*-acetylgalactosamine 4-sulfate 6-*o* sulfotransferase 15), *ZXDC* (ZXD family zinc finger C), *PDCD5* (programmed cell death 5), and LOC649548 (a pseudogene) (Fig. 1a). *DICER1* had lower expression in cases relative to controls (*F*-test, *P*=7.93E−06, genome-wide adjusted *P*=0.040, Fig. 1b).

Our *a priori* plan was to follow-up on the genes with the most promising evidence of being involved in PTSD and depression using a curated literature search approach. We decided to examine *DICER1* more closely for several reasons. First, *DICER1* is a conserved ribonuclease enzyme that processes precursor RNAs into mature short-interfering RNAs and miRNAs^9^,^12^, which regulates the expression of thousands of genes post-transcriptionally and involve in synaptic development, maturation and plasticity^13^,^14^. Second, *DICER1* expression was twofold reduced in the temporal cortex of patients with major depressive disorder relative to controls^7^. Third, regulation of expression of *DICER1* and miRNAs in the nucleus accumbens was found to be important in mediating vulnerability or resilience to stress in a chronic social defeat mouse model^8^. Lastly, studies have shown that decreasing *DICER1* expression in human peripheral cell lines stimulated the innate immune system via the upregulation of the major histocompatibility complex class I-related molecules^15^, and causal signatures for PTSD development was enriched for functions of the innate immune response in a prospective transcriptomic study^16^.

Next, since gene expression profiles can be affected by alcohol use, drug use, other psychiatric disorders or proportion of blood cell counts, we performed a secondary analysis in a subset of participants who had data on all of these variables. To that end, in a subset of 121 participants of the GTP discovery cohort, we performed genome-wide differential gene expression analysis between cases of PTSD&Dep and controls, adjusting simultaneously for sex, age, genotypic principal components, current alcohol and drug use disorders, bipolar disorder, current psychotic disorder and estimated cell counts. Consistent with our *DICER1* result from the primary analysis, we found that at genome-wide FDR<0.05, *DICER1* was significantly downregulated in cases compared with controls (*F*-test, *P*=0.0001, adjusted *P*=0.048), suggesting that the reduced expression of *DICER1* in the cases was not likely due to effects of drug or alcohol use, other psychiatric disorders or variation in immune cell proportions.

Subsequently we performed a validation of the downregulation of *DICER1* mRNA level in cases of PTSD&Dep compared with controls with quantitative PCR (qPCR) and found consistent results (1.3-fold downregulation in cases at *t*-test *P*=0.001). Since *DICER1* expression was significantly diminished in PTSD&Dep after sex, age, population substructure, alcohol and drug use, other psychiatric disorders and estimated blood cell count were adjusted for, we focused on *DICER1* expression, as reflected by the normalized intensity of the ILMN_1772692 probe, for follow-up studies. Hence, the genetic and imaging studies described in subsequent sections are targeted, hypothesis driven analyses to follow-up on our *DICER1* expression finding.

### *DICER1* SNPs versus *DICER1* expression in blood

**Figure 2:**
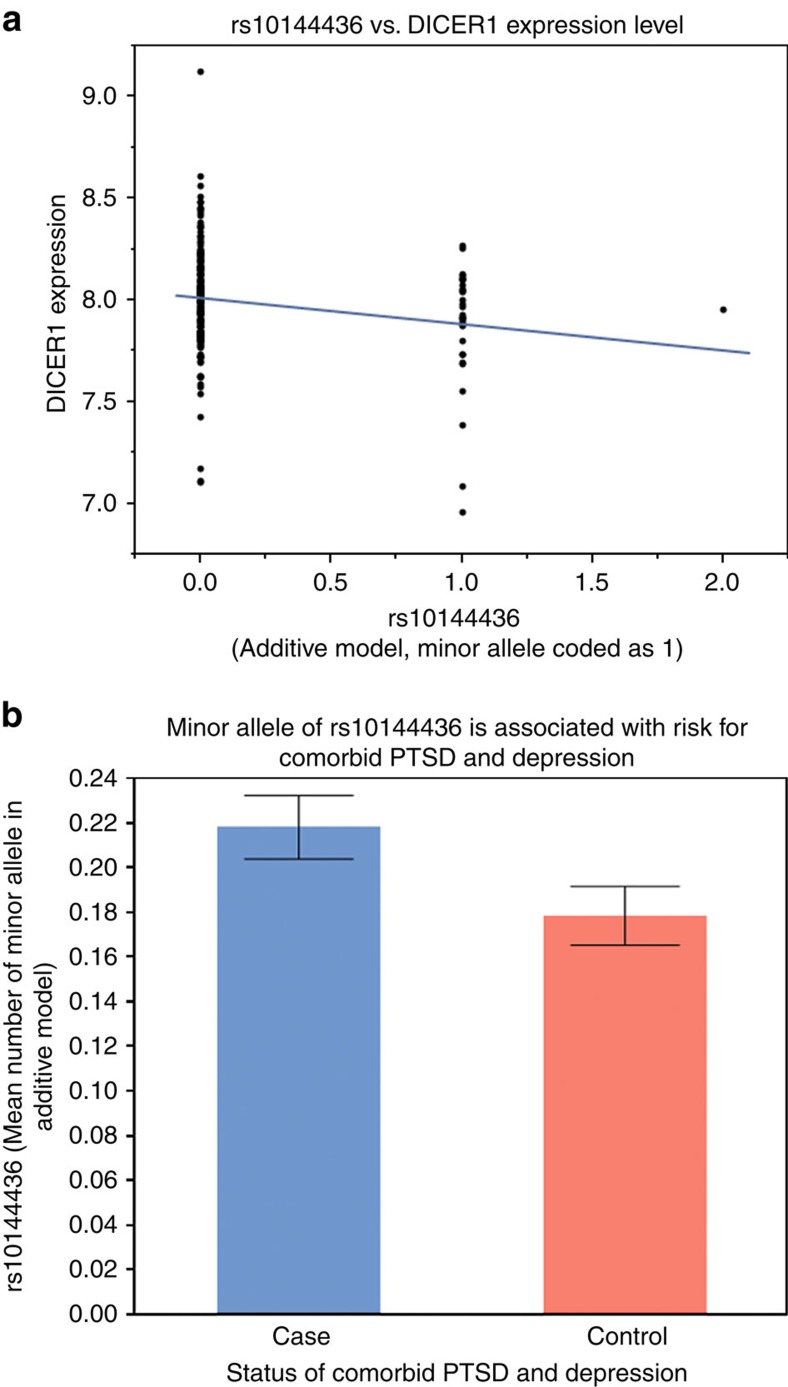
The SNP rs10144436, located in the 3′UTR of *DICER1*, is associated with *DICER1* mRNA expression level and with PTSD&Dep. (**a**) The minor allele of rs10144436 was associated with lower *DICER1* expression level. (**b**) The minor allele of rs10144436 was associated with PTSD&Dep. Of note the minor allele was coded as 1 in the additive model. Bar graph shows mean±s.e.m.

Given our finding of reduced *DICER1* expression in cases of PTSD&Dep versus controls, we next asked which single-nucleotide polymorphisms (SNPs) of the *DICER1* gene might influence its blood mRNA level. In our discovery GTP sample, the Illumina HumanOmni1-Quad BeadChip generated 14 *DICER1* tagging SNPs after standard genotype quality control as described in Methods. Out of these 14 SNPs, two were located in the coding regions, four in the 3′UTR and eight in the intronic regions. We found three SNPs significantly associated with *DICER1* expression level after controlling for sex and the principal components: rs10144436 (Wald test *P*=0.0034; *n*=191), rs1209904 (Wald test *P*=*P*=0.0018; *n*=191) and rs11160231 (Wald test *P*=0.0082; *n*=191). After we performed permutation to adjust for multiple testing^17^, only two SNPs remained significantly associated with *DICER1* expression: rs10144436 (*P*=0.0034; adjusted *P*=0.0452) and rs1209904 (*P*=0.0018; adjusted *P*=0.0277). These two eQTLs were in linkage disequilibrium with each other at *D*'=0.98 and *r*^2^=0.05. Specifically, rs1209904 is an intronic SNP and its minor allele (T) was associated with decreased *DICER1* expression. The other SNP, rs10144436, is located in the 3′UTR of *DICER1* and its minor allele (A) was also associated with lower *DICER1* mRNA level (Fig. 2a).

### *DICER1* eQTLs versus PTSD&Dep phenotype

Having identified two *DICER1* eQTL SNPs, rs10144436 (minor allele frequency (MAF)=0.098, Hardy–Weinberg equilibrium (HWE) *P* value=0.93, minor allele=A) and rs1209904 (MAF=0.337; HWE *P* value=0.03, minor allele=T), we next examined whether they were associated with PTSD&Dep in our 1876 all African American participants. Out of these two SNPs, only one, rs10144436, was significantly associated with PTSD&Dep after sex and population substructure via principal components were adjusted for (odds ratio=1.32, Wald test *P*=0.012, Bonferroni-adjusted *P*=0.024; *n*=1,874). Its minor allele (A) was associated with higher risk for PTSD&Dep. This is consistent with the expression finding, in which the minor allele of rs10144436 was associated with lower *DICER1* expression level, which was also associated with PTSD&Dep (Fig. 2b).

### Gene set enrichment analysis (GSEA) in PTSD&Dep

We next performed gene set enrichment analysis (GSEA)^18^ to gain insights into biological processes involving the differentially expressed genes in PTSD&Dep. Genes differentially expressed at *P* values<0.01 (*P* values ranged from 0.0098 to 7.92E−07) were included in the GSEA. We found many gene sets significantly enriched at FDR of 0.05 (Supplementary Table 1). Given the critical role of *DICER1* in the formation of mature miRNAs, it is notable that 13 sets of genes that were targets of various miRNAs and one set involving brain miRNA biogenesis in a mouse model of schizophrenia were significantly enriched, suggesting that genes that are targets of different miRNAs were differentially expressed in PTSD&Dep (Supplementary Table 1). Additionally, three gene sets related to the immune system were enriched: (a) genes involved in cytokine signalling, (b) genes upregulated in polymorphonuclear leukocytes after *Francisella tularensis* vaccination in human and (c) genes involved in the innate immune system (Supplementary Table 1).

### microRNA regulation in PTSD&Dep

Given the above GSEA results and our *DICER1* expression and genotype findings, which were replicated in three independent cohorts as described in the subsequent sections, we aimed to obtain more evidence towards our hypothesis that the *DICER1* and the miRNA pathway is involved in molecular mechanisms of PTSD&Dep by measuring blood miRNA levels in PTSD&Dep cases and controls. Using an RNA extraction protocol that preserves total RNA, we had 78 participants with available small RNAs and PTSD&Dep case or control status. We randomly selected 24 participants out of these 78 for a discovery sample, and the remaining 54 participants served as the replication sample. There was no difference in percentage of case status or percent of female between the 24 and 78 participants (Fisher Exact Test *P*=0.805 for percent of case status; *P*=0.800 for percent of female).

**Figure 3:**
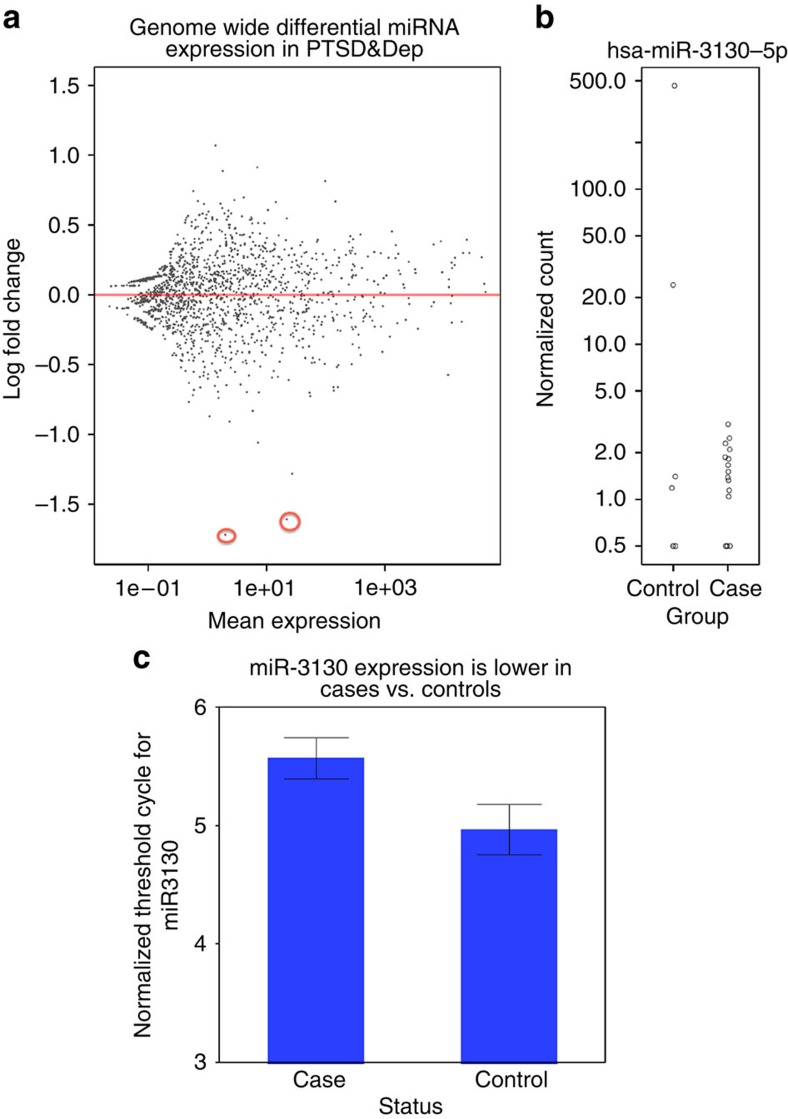
miRNA levels in PTSD&Dep cases vs. controls in the discovery (*n*=24) and replication sample (n=54). (**a**) MA plot for genome-wide differential expression of miRNAs in the discovery sample. The two points coloured in red are the two miRNAs with genome-wide adjusted *P* value<0.05 (miR-212-3p and miR-3130-5p). (**b**) miR-3130-5p expression was significantly lower in cases vs. controls in the discovery sample after sex, age and principal components were adjusted for. (**c**) miR-3130-5p expression was significantly downregulated in cases vs. controls in the replication sample after sex, age and genotypic principal components were adjusted for (*P*=0.030). Note, higher threshold cycle (*C*_T_) indicates lower abundance level. Error bar represents one s.e.m.

We next generated small RNA libraries for the discovery sample to perform small RNA sequencing. After standard quality control as described in Methods, we performed genome-wide differential miRNA expression analysis using DESeq2 (ref. 19), adjusting for sex, age and genotypic principal components. All 24 participants were African American, and there were 7 controls and 17 cases of PTSD&Dep. At genome-wide FDR<0.05, two miRNAs were significantly differentially expressed, and both were downregulated in the cases compared with the controls after sex, age and genotypic principal components were adjusted for: miR-212-3p (log_2_ fold change=−1.72; Wald test *P*=4.48E−05, adjusted *P*=0.048) and miR-3130-5p (log_2_ fold change=−1.61; Wald test *P*=4.97E−05, adjusted *P*=0.048; Fig. 3a,b).

The replication sample had 54 participants, all of whom were African American and 20 were controls and 34 were cases. Using qPCR, we examined the two miRNAs having adjusted *P*<0.05 above in the replication sample. We found that miR-3130-5p was significantly downregulated, with 1.57-fold downregulation, in the PTSD&Dep cases relative to controls after sex, age and genotypic principal components were adjusted for (*β*=0.284; χ^2^-test *P*=0.030; Fig. 3c). There was no significant association between miR-212-3p and PTSD&Dep in the replication sample. Of great interest, these two miRNAs have been studied in other neuropsychiatric disorders as described further in Discussion.

### *DICER1 and* miRNA regulation in PTSD&Dep

Using miRDB (miRDB.org), we identified 138 mRNAs that are potential targets of the miR-3130-5p at target score ⩾60 (list A, a.k.a Supplementary Table 2). From our original genome-wide differential gene expression analysis, 59 mRNAs were upregulated in the PTSD&Dep cases versus controls at FDR<0.1 (list B, a.k.a Supplementary Table 3). Since *DICER1* expression was reduced in the cases, we infer that levels of mature miRNAs in the cases would be reduced, and as the result, levels of their corresponding mRNA targets would be increased in the cases. The intersection of the list A (potential mRNA targets of miR-3130-5p) and list B (59 upregulated mRNAs in the cases) yielded one overlap: *MRPL35* (mitochondrial ribosomal protein L35). In this proof-of-concept study we found that in the cases of PTSD&Dep *DICER1* expression level was reduced, and consistently at least one mature miRNA had decreased abundance level, and one of its corresponding mRNA target had increased expression level relative to controls. Since miRNA-mediated gene silencing of targeted mRNA occurs through a combination of translational repression, mRNA deadenylation, decapping and decay^12^, it is not surprising that only a limited number of mRNA targets changed their abundance at the mRNA transcript level. In sum, this novel data support the notion that *DICER1* and the miRNA pathway is implicated in molecular mechanisms of PTSD&Dep.

### Neural correlates of *DICER1* gene expression

**Figure 4:**
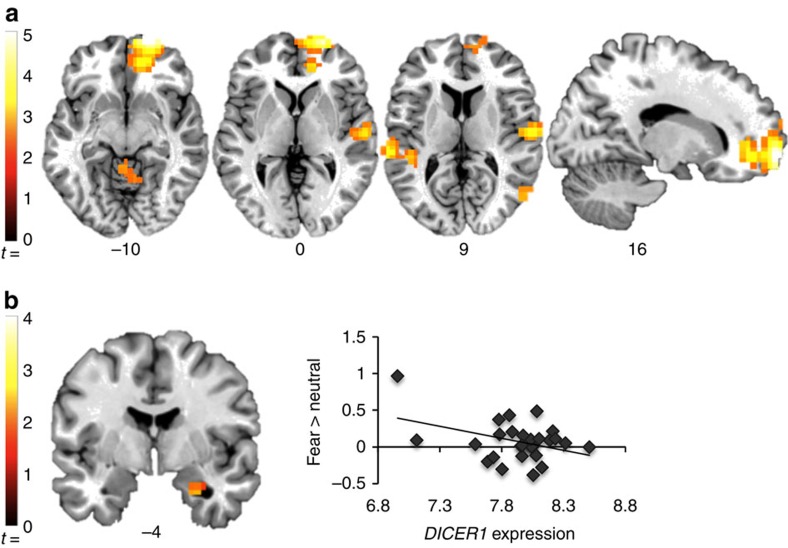
Lower *DICER1* expression in blood is significantly associated with more activation of the right amygdala in response to fearful faces, an intermediate phenotype for PTSD. Regions in which the response to threat (fearful>neutral face stimuli) correlated negatively with expression, *P*<0.05, corrected. (**a**) Clusters showing a significant correlation with expression in whole-brain analysis. Correction for multiple comparisons used a combined height-extent threshold calculated using Alphasim Monte-Carlo simulation, with 1,000 iterations and a cluster-forming threshold of *P*<0.01. (**b**) A significant negative correlation with expression in the amygdala region of interest (ROI). Amygdala ROI analyses used a small-volume correction implemented in Alphasim, with 1,000 iterations and a cluster-forming threshold of *P*<0.05. Scatter plot shows the average contrast estimate for the fear>neutral contrast, from voxels in the activated cluster. Images are shown in neurological orientation, overlaid on a single-subject template in Montreal Neurological Institute (MNI) space.

As our primary interest was to understand mechanisms underlying effects of *DICER1* expression on symptoms of PTSD and depression, we investigated how *DICER1* blood mRNA level might be related to neural responses to threat stimuli using fMRI. Specifically, we conducted a whole-brain analysis of linear correlation between *DICER1* expression levels and the contrast of fearful>neutral face stimuli in a subset of 28 traumatized women who provided both blood mRNA and fMRI data (Fig. 4, Table 2). Whole-brain analysis showed an inverse correlation between *DICER1* expression and activation in the right medial prefrontal cortex, bilateral temporal cortex, right posterior parietal cortex and cerebellum. No region correlated positively with *DICER1* expression levels. Since functional neuroimaging evidence has shown involvement of the amygdala, particularly its increased activation, in PTSD^20^,^21^, we selected this region for further examination. Amygdala region of interest analyses indicated a cluster in the right amygdala that correlated negatively with *DICER1* expression level (*x*, *y*, *z*=28, −4, −28; *Z*=2.19; *k*=11). No part of the amygdala showed a positive correlation with *DICER1* expression. In sum, we found that lower *DICER1* expression level was significantly associated with more amygdala activation to fearful stimuli, an intermediate phenotype for PTSD, which is consistent with our above finding of lower *DICER1* expression being associated with PTSD&Dep.

### Replication of reduced *DICER1* expression in PTSD&Dep

**Figure 5:**
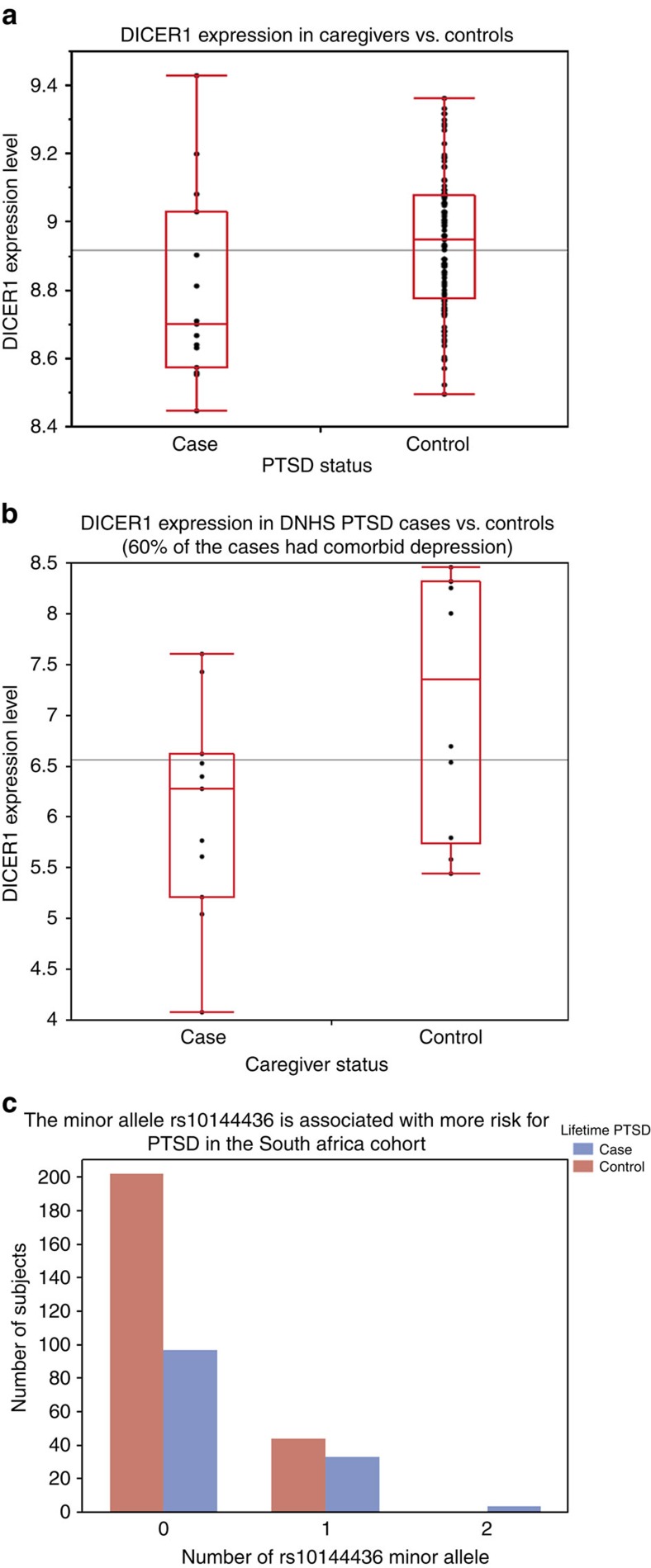
Replication of reduced *DICER1* expression level in. (**a**) The Detroit Neighborhood Health Study (DNHS) and (**b**) The Caregivers of family member with aggressive brain cancer (data from the publicly available data set GSE7893). The black horizontal lines represent the means of the overall samples. (**c**) Replication of the association between the *DICER1* eQTL rs10144436 and PTSD&Dep in the Drakenstein, South Africa cohort (minor allele was coded as 1 in the additive model).

We examined *DICER1* expression level in the Detroit Neighborhood Health Study (DNHS) cohort of 111 participants, out of whom 15 were cases of current PTSD (and 60% of these cases had comorbid current depression) and 96 were controls with no current PTSD and no current depression. Mean age among cases and controls was 48.5±12 and 54.7±15.4, proportion of female was 60% and 52%, percentage of African Americans was 66.7% and 86.5%, of European Americans was 33.3% and 12.5%, and of other ethnic group was 0 and 1.0%, respectively. Blood *DICER1* expression level, as reflected by normalized intensity levels of the ILMN_1772692 probe, was significantly reduced in cases relative to controls (*β*=−0.138, s.e.m.=0.062, *F*-test *P*=0.029) after sex, age and self-reported race were controlled for in a linear regression model (Fig. 5a). In a subset of 94 participants in whom we had principal components derived from genome-wide genotypes, *DICER1* expression continued to be significantly reduced in cases compared with controls after sex, age and principal components were adjusted for (*β*=−0.180, s.e.m.=0.072, *F*-test *P*=0.014). This *DICER1* expression finding is consistent with, and thus replicated, our finding of reduced *DICER1* blood expression level in cases of PTSD&Dep.

### Replication of reduced *DICER1* expression in another sample

We searched the publicly available datasets deposited in the Gene Expression Omnibus (GEO, in January of 2015) and found a relevant study on blood gene expression in a stressed cohort by Miller *et al*.^22^ (GSE7893). The authors investigated effects of severe psychological stress on transcriptional control pathways by surveying genome-wide differential gene expression profiles of caregivers of family members with aggressive brain tumours and matched controls^22^. The caregivers had significantly higher psychological distress, as reflected by higher perceived stress score (*t*-test *P*=0.003), decreased satisfaction with life (*t*-test *P*=0.04), and fewer positive emotions (*t*-test *P*<0.004) compared with controls^22^. Controls were matched for age, gender, ethnicity, marital status, cigarette and alcohol use, exercise, sleep habits, body mass index and history of cardiovascular diseases, as well as being free of major stressors such as divorce, bereavement, unemployment and family illness during the prior year^22^. Study subjects were recruited from the British Columbia in Vancouver. We downloaded the data and performed a secondary analysis on *DICER1* expression in this sample and found that *DICER1* expression was reduced in stressed cases versus controls (*β*=−0.54, *t*-test *P*=0.04, Fig. 5b). Further reading of the study publication confirmed our finding, as DICER1 was listed in Supplementary Table S1 in the supplemental data as underexpressed in stressed cases versus controls at an expression ratio of 0.47 and *P*<0.05. Assessment of PTSD was not done in this study; however, we think the severe, chronic and traumatizing stress caregivers experienced is arguably consistent with stress-related PTSD and depression. This finding is consistent with our finding of decreased *DICER1* expression in cases of PTSD&Dep.

### Replication of association between *DICER1* eQTL and PTSD&Dep

Here, we examined the association between the *DICER1* eQTL rs10144436 and PTSD&Dep in the Drakenstein, South African cohort. The Drakenstein cohort consists of pregnant women recruited from the peri-urban community of the Drakenstein subdistrict in South Africa. There is a high prevalence of trauma exposure in these at-risk women who were assessed longitudinally for trauma-related psychiatric disorders^23^. Post-traumatic stress disorder was assessed with the modified PTSD symptom scale (PSS)^24^ and the MINI^10^. Among the 380 participants with available genome-wide genotype markers from the PsychArray chip, there were 134 cases with lifetime PTSD and 246 controls with no lifetime PTSD. Among the 134 PTSD cases, ∼64% also had significant depression in their lifetime, as assessed by the MINI^10^ or having a score of ⩾14 on the Beck Depression Inventory (BDI)^25^,^26^. There were 60.4% Black and 39.6% mixed race among the cases and 56.1% Black and 43.9% mixed race among the controls. In this cohort, the *DICER1* rs10144436 had an MAF of 0.101, HWE *P* value of 0.82, and A as the minor allele. We found that rs10144436 was significantly associated with lifetime PTSD after population substructure was controlled for via principal components from genome-wide genotype markers (odds ratio=1.877, Wald test *P*=0.008, *n*=380). Consistent with our finding, the minor allele of rs10144436 was associated with increased risk for PTSD (Fig. 5c). Gene expression data are not available for this cohort.

## Discussion

Post-traumatic stress disorder with comorbid depression is highly prevalent among susceptible individuals following traumatic life events^6^. Here, we surveyed genome-wide differential gene expression profiles in blood in cases of PTSD&Dep and controls with no PTSD and no depression. We found that *DICER1* mRNA level was significantly reduced in the cases relative to the controls after we adjusted simultaneously for sex, age, population substructure, current alcohol and drug use disorders, current psychotic disorder, bipolar disorder and estimated blood cell counts. Importantly, downregulation of *DICER1* expression in PTSD&Dep was replicated in two independent cohorts, the DNHS^27^ and the Vancouver caregivers for family members with aggressive malignant brain cancer^22^. Consistently, our follow-up fMRI study found that lower *DICER1* blood mRNA level was significantly associated with increased amygdala activation to fearful stimuli, a neural correlate for PTSD. Furthermore, we found that a *DICER1* eQTL, rs10144436, located in its 3′UTR, was significantly associated with PTSD&Dep, and this association was replicated in a South African cohort of lifetime PTSD cases and controls^23^. Taken together, we infer that *DICER1* plays a role in the mechanism or manifestation of PTSD&Dep.

*DICER1* is a highly conserved RNAse III enzyme that is crucial for the biogenesis of noncoding, regulatory small RNAs, including miRNAs and short-interfering RNAs, in different tissues including the brain^8^,^9^,^12^. Specifically, *DICER1* converts precursor miRNAs to mature miRNAs, which not only fine-tune expression of thousands of genes via post-transcriptional repression in various tissues, including the nervous system but also involve in synaptic development, maturation and plasticity^13^,^14^. A growing number of studies have suggested the involvement of miRNAs in major depressive disorder, stress-related disorders and other psychiatric disorders^28^,^29^,^30^. Consistently, in our proof-of-concept study, we found that at least one miRNA, miR-3130-5p, had significantly reduced abundance level in the PTSD&Dep cases in both the discovery and replication samples, and one of its mRNA targets was upregulated in the PTSD&Dep cases versus controls after we controlled for sex, age and population substructure. Taken together, our findings support the notion that the *DICER1* and the miRNA pathway plays a role in the pathogenesis or manifestation of PTSD&Dep, potentially by regulating expression of the corresponding downstream mRNA targets and influencing cellular responses.

Of note, miR-3130 has been reported in other neuropsychiatric disorders. Specifically, a genome-wide association study of memory and hippocampal structure of 14,781 older adults found a genome-wide significant SNP that is located in the intronic region of the *FASTKD2* gene that is overlapped by miR-3130 (ref. 31). Although not replicated in our replication sample, miR-212 has been studied in Alzheimer's disease patients^32^. That study found that decreasing or increasing miR-212 expression in the temporal cortex and hippocampal neurons could induce apoptosis or neuroprotective effects respectively against oxidative stress^32^.

Stress-related *DICER1* and miRNA regulation in blood and brain may occur in parallel in human subjects, as our stress-related *DICER1* and miRNA findings in blood are consistent with stress-related *DICER1* and miRNA findings in the nucleus accumbens, a brain region involved in motivation, pleasure, reward, reinforcement learning and addiction, in a chronic social defeat stress mouse model^8^. In this study, Dias *et al*.^8^ found that β-catenin mediated vulnerability or resilience to stress through *DICER1* and miRNA regulation in the nucleus accumbens. Specifically, the authors found that *DICER1* was a robust target of β-catenin, that mice with *DICER1* knockdown (thus reduced or absent *DICER1* mRNA level) was easily susceptible to stress, and that in resilient mice, β-catenin turned on *DICER1* transcription^8^. Our finding is also consistent with another mouse study in which depletion of *DICER1* in the central amygdala led to an increase in anxiety-like behaviour, and that after acute restraint stress, expression profiles of miRNAs in the amygdala were changed^33^. Our findings are also in line with two human studies, in which *DICER1* expression was twofold reduced in the temporal cortex of patients with major depressive disorder relative to controls^7^, and global miRNA expression was reduced in the prefrontal cortex of depressed suicide completers relative to controls^34^.

Interestingly, *DICER1* mRNA level was found to be upregulated in lymphoblastoid cell lines of schizophrenia patients^35^ and also upregulated in the dorsolateral prefrontal cortex of schizophrenia patients^36^, suggesting the possibility of a positive correlation of *DICER1* expression between blood and brain; however, this remains to be investigated.

Given these findings, studies are needed to elucidate mechanisms linking the regulation of *DICER1* and miRNA in blood to that in the brain as well as to complex neurobehavioral phenotypes of PTSD and depression in humans. We hypothesize that this occurs via multiple pathways, and one potential pathway is the brain-immune interactions given the connection between *DICER1* expression and the innate immune system^15^,^37^,^38^. Specifically, decreased *DICER1* expression in human peripheral tissues (that is, embryonic kidney, dermal foreskin fibroblast or lung-tissue fibroblast) induces a DNA damage response, which stimulates the innate immune response by upregulation of the major histocompatibility complex class I-related molecules A and B^15^,^37^,^38^. The activated innate immune system creates a pro-inflammatory state, recruiting and activating neutrophils, and releasing cytokines from activated macrophages, among other processes^39^. A chronic inflammatory state, including elevated levels of pro-inflammatory cytokines or activated monocytes, has been shown to contribute to anxiety and depressive disorders by affecting metabolism of neurotransmitters in the brain or via monocyte trafficking to the brain in both mice and human studies^40^,^41^,^42^. Another potential mechanism is the regulation of the tightness or leakiness of the blood–brain barrier (BBB) by central or peripheral miRNAs^43^,^44^. A recent study of the mechanism of migration of breast cancer cells through the BBB in brain metastasis suggests that breast cancer cells with high metastatic capacity release extracellular vesicles containing proteins and miRNAs that can trigger the breakdown of the BBB^44^. Particularly, the study authors found that miR-181c in these peripheral tissue promotes the destruction of the BBB by regulating the actin dynamics^44^.

Prospective studies observed an increased risk of early-age cardiovascular disease, such as early-onset heart disease, stroke and myocardial ischaemia, in PTSD patients^45^,^46^. The involvement of *DICER1* and the miRNA pathway in PTSD is consistent with the overall psychosomatic picture of PTSD reported by these epidemiological studies, since both *DICER1* and miRNAs have been implicated in cardiovascular diseases. For instance, dysregulation of *DICER1*-dependent miRNA expression has been suggested to be a potential mechanism of impaired angiogenesis^47^ and impaired vascular remodelling^48^. Likewise, miRNAs have been shown to control important processes that contribute to cardiac hypertrophy, myocardial infarction, neovascularization, cardiac fibrosis, arrhythmia, angiogenesis and cholesterol regulation^49^,^50^.

Several studies have linked PTSD to increased activation of the amygdala to negative or fearful stimuli^20^,^21^. Here we found that decreased *DICER1* expression in blood was associated with greater amygdala reactivity to threat stimuli and to PTSD and depression symptom severity. Additionally, we found that diminished *DICER1* expression was associated with increased activation of the posterior parietal cortex, which engages in a host of cognitive functions including selective attention^51^. This is consistent with findings from another study, where war and torture survivors suffering from PTSD had hyperactivation of the superior parietal cortex selectively in response to aversive stimuli^52^. We also found an inverse correlation between *DICER1* expression and activation of the medial prefrontal cortex, which is not consistent with what is typically observed in PTSD^20^. However, this finding is consistent with hyperactivity of the medial prefrontal cortex during the self-referential processing of negative words in major depressed patients relative to controls^53^. Our fMRI sample included women having been exposed to traumatic experiences and developed high levels of PTSD and depression, and thus our neuroimaging *DICER1* findings are specific to this sample of participants; influence of *DICER1* expression on brain activation might be different in healthy participants. In future research, it would be interesting to investigate potential effects of *DICER1* expression on threat-processing circuitry using structural connectivity measures such as diffusion tensor imaging.

Our study has some limitations that should be taken into consideration when interpreting the results. Specifically, we did not have information on medications and tobacco use to control for in the gene expression analyses as they may influence gene expression profiles. We also did not assess for generalized anxiety disorder, social anxiety disorder or panic disorder to adjust for in the differential analysis. Additionally, the replication samples for *DICER1* expression level, the DNHS and caregiver samples, have relatively small sample sizes and thus higher potential for technical variation. Further, our genome-wide expression profiling of miRNAs was done in a small sample and thus likely lack the power to discover more miRNAs differentially regulated between PTSD&Dep cases and controls. A larger sample size is needed to discover more miRNAs differentially expressed in stress-related disorders such as PTSD or PTSD&Dep. Lastly, the observed differential expression of *DICER1* in PTSD&Dep could be reflective of an underlying genetic or epigenetic mechanism or a combination of both. Here, we presented evidence for an underlying genetic mechanism by showing a significant association between a polymorphism on the 3′UTR of *DICER1*, rs10144436, and PTSD&Dep. Even though this association remained significant after multiple testing corrections and was replicated in an independent South African cohort, it is worth mentioning that our finding still remains at the level of candidate gene analysis and not genome-wide association. Stronger evidence at the genome-wide association level is needed to confirm that a genetic mechanism underlies the association between *DICER1* expression and PTSD&Dep. It would be interesting for future studies to investigate the hypothesis that epigenetic mechanisms underlie the association between differential expression of *DICER1* and PTSD&Dep.

In summary, this is the first human study to our knowledge to suggest that *DICER1* and the miRNA regulation pathway are implicated in biological mechanisms of PTSD with comorbid depression. Studies are needed to elucidate the relationship between blood and brain *DICER1* and miRNA regulation as well as mechanisms underlying the connection between blood *DICER1* and miRNA regulation and stress-related psychiatric disorders to contribute to their prevention and treatment efforts.

## Methods

### Grady trauma project study participants

This study was part of a larger GTP investigating genetic and environmental factors contributing to PTSD in a population of inner city, low income and high stress and trauma exposed individuals. Inclusion criteria consisted of being 18 or older, understanding English and being able to give informed consent. Exclusion criteria included being acutely suicidal, psychotic or having acute medical problems. Participants gave informed consent, completed a battery of psychological measures and provided blood for genetic materials. This study has been approved by the Institutional Review Board of Emory University School of Medicine and Grady Memorial Hospital. A total of 184 men and women were included in this study based on availability of gene expression profiles and principal components derived from genome-wide genotype markers, and meeting the criteria for PTSD with comorbid depression (PTSD&Dep) as described in detail below. A subset of these participants was reported in a previous study focusing on childhood maltreatment and PTSD^54^.

### PTSD with comorbid depression (PTSD&Dep) phenotype

PTSD was assessed with the modified PSS, a psychometrically validated, 18-item self-report scale with excellent internal consistency, high test-retest reliability and concurrent validity, as well as mirroring the DSM-IV criteria for PTSD^24^. A PSS cutoff score of 14 yields a sensitivity of 91% and specificity of 62% for identifying PTSD when compared with the diagnosis of PTSD made by the clinician-administered PTSD scale in 229 motor vehicle accident survivors^55^. Hence, we operationally defined PSS scores ⩾14 to indicate clinically significant current PTSD symptoms.

Depressive symptoms were measured with the psychometrically validated 21-item BDI^25^. The BDI has an excellent internal consistency (reliability coefficient of 0.93) and is commonly used in depression research studies^25^. Among African American primary care patients, a BDI cutoff score of 14 yielded a sensitivity of 88% and specificity of 84% when compared with a diagnosis of MDD made by PRIME-MD Mood Module, a focused interview for MDD using DSM-IV criteria^26^. Given these data and that 94% of our study participants are African Americans, we used a BDI score ⩾14 to indicate clinically significant current depression.

Cases of current PTSD&Dep were defined as having both a PSS⩾14 and BDI⩾14. Controls were defined as not having either significant PTSD or depression, as reflected by a PSS ≤7 and BDI ≤7. There were 112 cases of current PTSD&Dep and 72 controls in this study.

### Other psychiatric disorders

Current alcohol or drug use disorder, bipolar disorder and current psychotic disorder were assessed using the SCID^11^ or MINI^10^, and was available in a subset of 145–150 participants.

### Gene expression profiles in the GTP cohort

RNA was extracted from whole blood collected in Tempus tubes at about 0830, h for all participants. All samples had Bioanalyzer RNA Integrity Number (RIN) ⩾6. Complementary DNA was derived from RNA and then hybridized, and raw probe intensities were generated on Illumina HumanHT-12 v3 or v4 BeadChip arrays. Probes with detection *P* value>0.01 in at least 95% individuals were filtered out, leaving 15,877 probes from the v3 chip and 17,048 probes from the v4 chip. Combining probes from v3 and v4 chips yielded 13,048 probes in common to be included in the analysis. We then performed log_2_ transformation and normalization of probe intensities using the Supervised Normalization of Microarray algorithm, removing the effects of batch and RIN^56^. The gene expression data set has been deposited at the Gene Expression Omnibus with the accession number GSE67663.

### Genotypes and principal components in the GTP cohort

DNA was extracted from saliva or blood, and genome-wide SNP genotyping was conducted using Illumina's HumanOmni1-Quad or OmniExpress BeadChips. Standard quality control of the genome-wide data was performed using PLINK^57^, removing individuals with greater than 2% missing data and removing one in each pair of related individuals with an identity by descent proportion>0.12 (indicating cousins or a closer relation). We removed SNPs with call rates ≤ 98%, MAF<0.01, and HWE *P* value<1 × 10^−6^ in PTSD controls. After restricting the SNPs to the autosomes, we used PLINK to prune the data in windows of 50 base pairs, removing one SNP from each pair of SNPs with *r*^2^>0.05 to obtain a set of roughly independent SNPs. This reduced set of SNPs was used in principal component analysis to infer axes of ancestry and remove outlier subjects^58^. The gene expression analysis utilized the principal components generated from combined genome-wide data from the two chips (HumanOmni1-Quad and OmniExpress) on the subset of subjects with expression data. The candidate gene analysis was conducted using *DICER1* SNPs genotyped on the HumanOmni1-Quad Beadchip only, with similar quality control as described above (African American only, *n*=1,874 meeting definition of PTSD&Dep cases and controls).

### Genome-wide differential gene expression analysis in GTP cohort

Genome-wide differential gene expression between cases of current PTSD&Dep and controls was surveyed using analysis of covariance, in which normalized gene expression level for each probe was the outcome, PTSD&Dep the dichotomous independent variable, sex, age and genotypic principal components as the covariates. Age was categorized into three groups, below 33rd percentile, from 33rd to 67th percentile and above 67th percentile. The first five principal components, described above, were used as covariates to control for population substructure. Multiple testing was accounted for with the Benjamini and Hochberg FDR of 0.05 (ref. 59). Analyses were performed using JMP Genomics version 7.

### GSEA

To gain insight into biological processes involving the differentially expressed genes between cases and controls, we performed GSEA using the pre-ranked GSEA analytical software from the Broad Institute^18^. Genes that were differentially expressed between cases and controls at genome-wide FDR<0.20 (that is, genes with nominal *P* values ranging between 5.97E−07 and 0.0098, *n*=658 genes) were pre-ranked using their *t*-statistics derived from the analysis of covariance for differential expression between cases and controls as described above. Gene sets were obtained from the Molecular Signature Database (MSigD) v4.0 (ref. 18). We selected the gene sets from the curated, gene ontology, positional, motif, computational, oncogenic and immunologic signatures collections, particularly those with at least 15 genes in common with our pre-ranked gene list, and the weighted *p* option (*P*=1) for the running sum statistics for enrichment score. Pre-ranked GSEA assessed the significance for each gene set using 5000 permutations of case and the control status for each set, and multiple hypothesis testing was adjusted with Benjamini and Hochberg FDR^18^,^59^.

### Cell count estimate

Cell type proportions were estimated from genome-wide DNA methylation data from blood samples drawn at the same visit as that used for the expression arrays. The proportions of granulocytes and lymphocytes in each sample were estimated using publically available data (GSE36069) and the method described by Houseman *et al*.^60^,^61^.

### qPCR validation of DICER1 differential expression in PTSD&Dep

In a subset of 40 subjects (20 cases of PTSD&Dep and 20 controls), whose RNA was run on the Illumina HumanHT12 Beadchip arrays, total RNA from whole blood was reverse transcribed into cDNA using the RT2 First Strand Kit (Qiagen). *DICER1* and *GAPDH* as housekeeping control were detected and quantified in duplicates using the TaqMan assays Hs00229023_m1 (*DICER1*) and Hs02758991_g1 (*GAPDH*) with TaqMan Gene Expression Master Mix on a ViiA7 real-time PCR system in 384-well format according to the instruction of the manufacturer (Applied Biosciences). Gene expression differences and fold changes were calculated using the 2^−ΔΔCt^ method^62^.

### Candidate gene and eQTL analyses in the GTP cohort

Associations between *DICER1* SNPs and *DICER1* expression levels were performed in PLINK using the linear regression model, in which normalized *DICER1* expression level was the outcome, SNP genotype the independent variable and sex and the first five principal components as covariates. We used the additive model for SNP genotype, with the minor allele coded as 1. Multiple testing was addressed with permutation^17^. Analyses for association between *DICER1* tagging SNPs and PTSD&Dep were performed in PLINK using logistic model, in which status of PTSD&Dep was the outcome, SNP genotype the independent variable and sex and the first five principal components as covariates. Likewise, we used the additive model for the SNP genotype, with the minor allele coded as 1, and addressing multiple testing with permutation^17^.

### Small RNA sequencing and analysis in the GTP discovery sample

Total RNA was extracted from whole blood, which was collected in Tempus tubes at 0830, h for all participants, following the Norgen protocol (Norgen Biotek Inc.). Small RNA was size fractionated from total RNA using polyacrylamide gel and then used as the starting material to generate small RNA libraries following the Illumina TruSeq Small RNA protocol. The 24 small RNA libraries were then multiplexed and sequenced single-end reads on two lanes of the Illumina HiSeq1000.

Quality of the sequencing data was inspected with FastQC (bioinformatics.babraham.ac.uk/projects/fastqc/). Adapters were trimmed with cutadapt (https://cutadapt.readthedocs.org/en/stable/), and after trimming, sequences shorter than 14 nucleotides were discarded. Read length distribution after adapter trimming was visualized and observed to be centred at 22 nucleotides, indicating high-quality miRNA-seq library preparation^63^. Trimmed reads were aligned to the miRBase v.21 (ref. 64) with SHRiMP aligner ((http://compbio.cs.toronto.edu/shrimp/), and only reads with a minimum average PHRED score of 10 were considered for alignment. Percent reads mapped to mature miRNAs ranged from 85 to 95%, indicating high-quality libraries and sequencing data. The pipeline above was organized by the miRNA analysis application^65^. After removing an outlier sample with an extremely low raw count and four miRNAs with extremely high counts, genome-wide differential miRNA analysis was performed using DESeq2 (ref. 19), adjusting for sex, age and five genotypic principal components. Age was categorized into three groups, upper third, middle third and lower third. Multiple testing was addressed with Benjamini and Hochberg FDR^19^,^59^.

### miRNA qPCR assays in the GTP replication sample (*n*=54)

Total RNA extracted from whole blood was reverse transcribed into cDNA using Qiagen miScript II RT kits and protocol. Hsa-miR-212-3p, hsa-miR-3130-5p and snRNA RNU6B as housekeeping control were detected and quantified in triplicates using Qiagen miScript Primer Assays Hs_miR-212_1, Hs_miR-3130-5p_1 and Hs_RNU6-2_11 with miScript SYBR Green PCR kit on a ViiA7 real-time PCR system in 384-well format according to the instruction of the manufacturer (Qiagen). Normalized threshold cycle (*C*_T_) values and fold change for miR-212-3p and miR-3130-5p were calculated using the 2^−ΔΔCt^ method^62^. Differential expression analysis for each of the two miRNAs was performed using a generalized linear model in JMP Genomics, in which the normalized *C*_T_ value was the outcome, PTSD&Dep status the independent variable, and sex, age, and five genotypic principal components as the covariates.

### Functional MRI study in the GTP cohort

We examined neural correlates of *DICER1* expression in a functional MRI study among a subset of traumatized women (*n*=28) for whom we had both mRNA and fMRI data, drawn from the larger GTP sample. Participants completed a task designed to engage threat-processing networks via passively viewing static fearful and neutral face stimuli. Blocks of either eight fearful or eight neutral face stimuli selected from the stimulus set of Ekman and Friesen^66^ were presented in a pseudo-random order, with each stimulus presented for 500 ms. These fearful and neutral stimuli were sorted in random order for each block. Participants were instructed to pay attention to the faces but not make any behavioural responses to minimize motion artefacts, neural activation unrelated to the processing of the visual stimulus, or dampening of emotional arousal^67^. Brain imaging data were acquired on a Siemens 3.0 Tesla Magnetom Trio TIM whole-body MR Scanner using a 12-channel head coil. Pre-processing and statistical analyses were performed using Statistical Parametric Mapping software (SPM5, Wellcome Trust Centre for Neuroimaging) and AFNI (http://afni.nimh.nih.gov/afni/). Statistical contrasts between conditions (for example, fearful versus neutral) were assessed with linear contrasts. First-level models included fearful and neutral task conditions and six motion parameters. Second-level models were conducted as a correlation between *DICER1* expression levels (continuous variable) and the contrast of fearful>neutral. Correction for multiple comparisons was through a combined height-extent threshold calculated using Alphasim Monte-Carlo simulation with 1,000 iterations and a cluster-forming threshold of *P*<0.01 (ref. 68).

### Detroit neighborhood health study (DNHS)

In the DNHS cohort structured telephone interviews were conducted to assess PTSD (with the PTSD Checklist Civilian Version (PCL-C)) and depression (with the Patient Health Questionnaire (PHQ-9))^69^. Post-traumatic stress disorder and depression diagnoses obtained from the telephone interview responses have been validated in a random subsample of participants via in-person clinical interview using the clinician-administered PTSD scale and Structured Clinical Interview for DSM-IV Disorders, and the comparison showed high internal consistency and concordance^69^.

Trained phlebotomists conducted venipuncture blood draws during scheduled home visits to consenting participants. Following the blood draw, all blood samples were sent to Wayne State University in Detroit, Michigan for processing. RNA was isolated from leukocytes using Leukolocks kits (Ambion, Austin, TX) following the manufacturer's alternative protocol to preserve total RNA. All included samples had RIN ⩾5, 28 s/18 s⩾1.0 and 260/280 ⩾1.7. Illumina HT-12 V4 Expression BeadChip was used to generate raw expression data, which was processed using the Bioconductor (http://bioconductor.org) Limma and ComBat packages to background correct, normalize and adjust for batch effects. The data was filtered to remove any low or unexpressed probes; after filtration, 28,352 probes remained.

In a subset of 94 participants (out of 111) in this cohort we had principal components derived from genome-wide genotypes. Participants' DNA was isolated from whole blood or saliva, and genome-wide genotypes were obtained using the Illumina HumanOmniExpress BeadChips. A total of 688,890 SNPs passed quality control filters, including call rate>95%, MAF>0.01, and HWE *P*>1 × 10^−6^. The Multidimensional scaling analysis of genome-wide identity-by-state data implemented in PLINK was used to determine ancestry in the whole sample. The first two components from the multidimensional scaling analysis distinguished African American from European American participants and others, and the second component distinguished Hispanic from non-Hispanic participants^70^. We therefore used the first two principal components to adjust for population substructure in the *DICER1* expression replication analysis.

### Drakenstein, South Africa cohort genotyping

Genome-wide genotype markers for this cohort were obtained using the Illumina Infinium PsychArray Beadchip. Quality control of the genotypes was performed to exclude individuals with>5% missing data, SNPs with call rates<95%, SNPs with MAF<0.05, and SNPs with HWE *P* value<1 × 10^−6^ in controls and<1 × 10^−10^ in PTSD cases, as well as removing duplicates and one in each pair of cryptically related individuals who had an identity by descent proportion>0.12 (indicating cousins or a closer relation). Principal components were created using 40,000 random and independent genome-wide SNPs. As only the first principal component had the Tracy–Widom test *P* value<0.05, it was used as a covariate in the association analysis to adjust for population substructure. *DICER1* SNP rs10144436 was extracted from the above genotypes to perform association analysis with lifetime PTSD in PLINK using logistic model, co-varying for the first principal component. All participants were women (*n*=380).

## Additional information

**Accession codes:** The gene expression data and small RNA sequencing data have been deposited at the Gene Expression Omnibus with the accession numbers GSE67663 and GSE74162 respectively.

**How to cite this article:** Wingo, A. P. *et al*. *DICER1* and microRNA regulation in post-traumatic stress disorder with comorbid depression. *Nat. Commun.* 6:10106 doi: 10.1038/ncomms10106 (2015).

## Supplementary Material

Supplementary Information Supplementary Tables 1-3.

## Figures and Tables

**Table 1:** Characteristics of the GTP discovery sample of cases of PTSD&Dep and controls with no PTSD and no depression (*n*=184).

**Characteristics**	**Cases (*****n*****=112)**	**Controls (*****n*****=72)**	***P***-value*
Age	41.9±10.7	43.3±14.9	0.28
Sex (% female)	77.7%	70.8%	0.29
Race (% African American)	91.1%	98.6%	0.04
PSS† score	28.7±9.2	2.1±2.3	<0.0001
BDI‡ score	28.2±10.1	2.7±2.1	<0.0001
Current substance use disorder§ (yes)	18.0%	9.8%	0.17
Current psychotic disorder|| (yes)	16.3%	6.8%	0.12
Bipolar disorder¶ (yes)	2.3%	0.0%	0.51
Number of traumatic experiences	7.3±3.8	3.4±2.8	<0.0001

BDI; Beck Depression Inventory; GTP, Grady Trauma Project; MINI, Mini International Neuropsychiatric Interview; PSS, PTSD symptom scale; PTSD, post-traumatic stress disorder; PTSD&Dep, PTSD with comorbid depression; SCID, Structure Clinical Interview for DSM-IV.

^*^P values were from Wilcoxon Rank Sums or Fisher exact test.

^†^PSS: PTSD symptom severity score from the PSS.

^‡^BDI: depression severity score from the BDI.

^§^Available in a subset of 150 subjects; assessed by the SCID and MINI.

^||^Available in a subset of 145 subjects; assessed by the SCID and MINI.

^¶^Available in a subset of 148 subjects; assessed by the SCID and MINI.

**Table 2:** Whole-brain analysis of linear correlation between *DICER1* expression level and the contrast of fearful>neutral face stimuli in 28 female participants.

		**MNI coordinates**				
**Region**	**HEM**	***x***	***y***	***z***	***Z***	***k***
*Positive correlation with DICER1 expression*						
No significant clusters						
*Negative correlation with DICER1 expression*						
Sup. frontal G.	R	20	68	−8	4.27	211
Orbitofrontal G.	R	16	44	−8	3.23	(LM)
Sup. frontal G.	R	12	52	−24	2.56	(LM)
Sup. temporal G.	R	60	−16	4	3.27	85
Sup. temporal G.	R	68	−28	20	3.01	(LM)
Supramarginal G.	R	64	−20	44	2.9	(LM)
Mid. temporal G.	L	−64	−32	8	3.17	53
Sup. temporal G.	L	−44	−36	8	2.77	(LM)
Sup. temporal G.	L	−48	−40	20	2.45	(LM)
Mid. temporal G.	R	56	−68	12	2.91	22
Angular G.	R	48	−68	44	3	27
Angular G.	R	48	−72	36	2.97	(LM)
Cerebellum	L	−4	−44	−8	2.59	24
Cerebellum	L	8	−52	−8	2.48	(LM)

MNI, Montreal Neurological Institute; HEM, Hemisphere; LM, Local Maximum.
