# Corrigendum: *DICER1* and microRNA regulation in post-traumatic stress disorder with comorbid depression

**DOI:** 10.1038/ncomms10958

**Published:** 2016-03-03

**Authors:** Aliza P. Wingo, Lynn M. Almli, Jennifer S. Stevens, Torsten Klengel, Monica Uddin, Yujing Li, Angela C. Bustamante, Adriana Lori, Nastassja Koen, Dan J. Stein, Alicia K. Smith, Allison E. Aiello, Karestan C. Koenen, Derek E. Wildman, Sandro Galea, Bekh Bradley, Elisabeth B. Binder, Peng Jin, Greg Gibson, Kerry J. Ressler

Nature Communications 6: Article number: 10106 10.1038/ncomms10106 (2015); Published: 12032015; Updated: 03032016

The original version of this Article contained an error in the spelling of the author Jennifer S. Stevens, which was incorrectly given as Jennifer J. Stevens. Furthermore, in Fig. 5, the titles for panels a and b were inadvertently switched. Both of these errors have now been corrected in the PDF and HTML versions of the Article.

