# Supplementary material for: DICER1 and microRNA regulation in post-traumatic stress disorder with comorbid depression
Source: Nat Commun. 2015 Dec 3;6:10106. doi: 10.1038/ncomms10106 (PMC4686835; doi:10.1038/ncomms10106)
Supplement: Supplementary Information — Supplementary Tables 1-3. [file ncomms10106-s1.pdf]

Supplementary Table 1: Selected gene sets significantly enriched at FDR<0.05 among the genes differentially expressed between cases of PTSD&Dep and controls (at *p*<0.01) from GSEA.

**Description of gene set**	**NES**	**nominal *p***	**FDR *q*-value**
microRNA related			
Targets of microRNA GTGCCTT, MIR-506	-2.94	<2.00E-4	5.31E-5
Targets of microRNA TGCTGCT, MIR-15A, MIR-16, MIR-15B, MIR-195, MIR-424, MIR-497	-2.84	<2.00E-4	1.02E-4
Targets of microRNA TTTGCAC, MIR-19A, MIR-19B	-2.60	<2.00E-4	4.71E-4
Targets of microRNA TGGTGCT, MIR-29A, MIR-29B, MIR-29C	-2.44	<2.00E-4	1.19E-3
Targets of microRNA AATGTGA, MIR-23A, MIR-23B	-2.34	<2.00E-4	2.09E-3
Targets of microRNA CTCAGGG, MIR-125B, MIR-125A	-2.33	4.08E-4	2.21E-3
Targets of microRNA TGAATGT, MIR-181A, MIR-181B, MIR-181C, MIR-181D	-2.31	1.63E-3	2.47E-3
Targets of microRNA TTGCCAA, MIR-182	-2.26	2.82E-3	3.45E-3
Targets of microRNA GCACTTT, MIR-17-5P, MIR-20A, MIR-106A, MIR-106B, MIR-20B, MIR-519D	-2.20	1.60E-3	4.76E-3
Targets of microRNA CTTTGTA, MIR-524	-2.15	1.65E-3	6.23E-3
Targets of microRNA TTGCACT, MIR-130A, MIR-301, MIR-130B	-2.13	2.41E-3	6.77E-3
Targets of microRNA TGCACTT, MIR-519C, MIR-519B, MIR-519A	-2.12	2.02E-3	7.32E-3
Targets of microRNA ACTTTAT, MIR-142-5P	-2.07	3.23E-3	9.61E-3
Genes involved in brain microRNA biogenesis in mouse model of schizophrenia	3.07	<2.00E-4	0.00
Immune system related			
Genes up-regulated in polymorphonuclear leukocytes after *Francisella tularensis* vaccine in human	-2.58	<2.00E-4	6.66E-4
Genes involved in cytokine signaling in the immune system	-2.46	3.99E-4	1.14E-3
Genes involved in the innate immune system	-2.44	<2.00E-4	1.20E-3

NES: normalized enrichment score. FDR: false discovery rate

Supplementary Table 2: list A of 138 mRNA that are potential targets of the miR-3130-5p at target score ≥60

Target Rank	Target Score	miRNA Name	Gene ID	Gene Symbol	Transcript Accession
1	97	hsa-miR-3130-5p	23608	MKRN1	NM_013446
2	96	hsa-miR-3130-5p	51062	ATL1	NM_181598
3	96	hsa-miR-3130-5p	5495	PPM1B	NM_177969
4	95	hsa-miR-3130-5p	79183	TTPAL	NM_001039199
5	95	hsa-miR-3130-5p	2045	EPHA7	NM_004440
6	94	hsa-miR-3130-5p	80351	TNKS2	NM_025235
7	94	hsa-miR-3130-5p	3839	KPNA3	NM_002267
8	93	hsa-miR-3130-5p	80856	KIAA1715	NM_030650
9	93	hsa-miR-3130-5p	7468	WHSC1	NM_133331
10	91	hsa-miR-3130-5p	41	ASIC1	NM_020039
11	91	hsa-miR-3130-5p	51552	RAB14	NM_016322
12	89	hsa-miR-3130-5p	1184	CLCN5	NM_001282163
13	89	hsa-miR-3130-5p	57135	DAZ4	NM_020420
14	89	hsa-miR-3130-5p	57054	DAZ3	NM_020364
15	89	hsa-miR-3130-5p	57055	DAZ2	NM_001005786
16	89	hsa-miR-3130-5p	1617	DAZ1	NM_004081
17	88	hsa-miR-3130-5p	284615	ANKRD34A	NM_001039888
18	88	hsa-miR-3130-5p	7750	ZMYM2	NM_003453
19	88	hsa-miR-3130-5p	51542	VPS54	NM_001005739
20	88	hsa-miR-3130-5p	401265	KLHL31	NM_001003760
21	87	hsa-miR-3130-5p	83897	KRTAP3-2	NM_031959
22	87	hsa-miR-3130-5p	84920	ALG10	NM_032834
23	87	hsa-miR-3130-5p	56172	ANKH	NM_054027
24	87	hsa-miR-3130-5p	129881	CCDC173	NM_001085447
25	86	hsa-miR-3130-5p	10166	SLC25A15	NM_014252
26	86	hsa-miR-3130-5p	5007	OSBP	NM_002556
27	85	hsa-miR-3130-5p	60526	C2orf43	NM_021925
28	85	hsa-miR-3130-5p	6879	TAF7	NM_005642
29	85	hsa-miR-3130-5p	54764	ZRANB1	NM_017580
30	85	hsa-miR-3130-5p	57700	FAM160B1	NM_020940
31	85	hsa-miR-3130-5p	22858	ICK	NM_016513
32	84	hsa-miR-3130-5p	9705	ST18	NM_014682
33	84	hsa-miR-3130-5p	54700	RRN3	NM_018427
34	83	hsa-miR-3130-5p	83394	PITPNM3	NM_001165966
35	83	hsa-miR-3130-5p	3171	FOXA3	NM_004497
36	83	hsa-miR-3130-5p	3916	LAMP1	NM_005561
37	83	hsa-miR-3130-5p	63894	VIPAS39	NM_022067
38	83	hsa-miR-3130-5p	66008	TRAK2	NM_015049
39	83	hsa-miR-3130-5p	5887	RAD23B	NM_001244724

40	83	hsa-miR-3130-5p	56829	ZC3HAV1	NM_020119
41	82	hsa-miR-3130-5p	55041	PLEKHB2	NM_001267065
42	82	hsa-miR-3130-5p	29933	GPR132	NM_001278694
43	81	hsa-miR-3130-5p	143384	CACUL1	NM_153810
44	81	hsa-miR-3130-5p	151050	KANSL1L	NM_152519
45	81	hsa-miR-3130-5p	5420	PODXL	NM_001018111
46	80	hsa-miR-3130-5p	93986	FOXP2	NM_148898
47	80	hsa-miR-3130-5p	80830	APOL6	NM_030641
48	79	hsa-miR-3130-5p	80335	WDR82	NM_025222
49	79	hsa-miR-3130-5p	253635	GPATCH11	NM_174931
50	79	hsa-miR-3130-5p	4613	MYCN	NM_005378
51	79	hsa-miR-3130-5p	51104	ABHD17B	NM_001025780
52	79	hsa-miR-3130-5p	6323	SCN1A	NM_006920
53	79	hsa-miR-3130-5p	6932	TCF7	NM_201634
54	78	hsa-miR-3130-5p	2900	GRIK4	NM_001282470
55	78	hsa-miR-3130-5p	348110	C15orf38	NM_182616
56	78	hsa-miR-3130-5p	57489	ODF2L	NM_001184766
57	78	hsa-miR-3130-5p	51473	DCDC2	NM_001195610
58	78	hsa-miR-3130-5p	8322	FZD4	NM_012193
59	78	hsa-miR-3130-5p	113201	CASC4	NM_138423
60	77	hsa-miR-3130-5p	51163	DBR1	NM_016216
61	77	hsa-miR-3130-5p	355	FAS	NM_152871
62	76	hsa-miR-3130-5p	140739	UBE2F	NM_001278308
63	76	hsa-miR-3130-5p	7516	XRCC2	NM_005431
64	76	hsa-miR-3130-5p	100101467	ZSCAN30	NM_001288711
65	75	hsa-miR-3130-5p	89795	NAV3	NM_014903
66	75	hsa-miR-3130-5p	85013	TMEM128	NM_032927
67	75	hsa-miR-3130-5p	26046	LTN1	NM_015565
68	75	hsa-miR-3130-5p	339500	ZNF678	NM_178549
69	75	hsa-miR-3130-5p	6672	SP100	NM_003113
70	74	hsa-miR-3130-5p	56889	TM9SF3	NM_020123
71	74	hsa-miR-3130-5p	6605	SMARCE1	NM_003079
72	74	hsa-miR-3130-5p	891	CCNB1	NM_031966
73	73	hsa-miR-3130-5p	115350	FCRL1	NM_001159398
74	73	hsa-miR-3130-5p	83445	GSG1	NM_001206843
75	73	hsa-miR-3130-5p	54883	CWC25	NM_017748
76	73	hsa-miR-3130-5p	8445	DYRK2	NM_006482
77	72	hsa-miR-3130-5p	445571	CBWD3	NM_201453
78	72	hsa-miR-3130-5p	101060578	LOC101060578	XM_006726501
79	72	hsa-miR-3130-5p	644019	CBWD6	NM_001085457
80	72	hsa-miR-3130-5p	374928	ZNF773	NM_198542
81	72	hsa-miR-3130-5p	8464	SUPT3H	NM_181356
82	72	hsa-miR-3130-5p	5565	PRKAB2	NM_005399

83	72	hsa-miR-3130-5p	79085	SLC25A23	NM_024103
84	72	hsa-miR-3130-5p	55871	CBWD1	NM_001145355
85	72	hsa-miR-3130-5p	6096	RORB	NM_006914
86	72	hsa-miR-3130-5p	150472	CBWD2	NM_172003
87	72	hsa-miR-3130-5p	220869	CBWD5	NM_001286836
88	71	hsa-miR-3130-5p	246243	RNASEH1	NM_001286834
89	71	hsa-miR-3130-5p	84285	EIF1AD	NM_032325
90	71	hsa-miR-3130-5p	488	ATP2A2	NM_170665
91	71	hsa-miR-3130-5p	6480	ST6GAL1	NM_003032
92	70	hsa-miR-3130-5p	5229	PGGT1B	NM_005023
93	70	hsa-miR-3130-5p	26065	LSM14A	NM_015578
94	69	hsa-miR-3130-5p	6492	SIM1	NM_005068
95	69	hsa-miR-3130-5p	55660	PRPF40A	NM_017892
96	69	hsa-miR-3130-5p	51318	MRPL35	NM_145644
97	69	hsa-miR-3130-5p	30000	TNPO2	NM_001136196
98	69	hsa-miR-3130-5p	1016	CDH18	NM_001167667
99	68	hsa-miR-3130-5p	7257	TSNAX	NM_005999
100	68	hsa-miR-3130-5p	115509	ZNF689	NM_138447
101	68	hsa-miR-3130-5p	3038	HAS3	NM_005329
102	68	hsa-miR-3130-5p	9750	FAM65B	NM_014722
103	67	hsa-miR-3130-5p	1562	CYP2C18	NM_000772
104	67	hsa-miR-3130-5p	3329	HSPD1	NM_002156
105	67	hsa-miR-3130-5p	5000	ORC4	NM_001190879
106	66	hsa-miR-3130-5p	101927658	GOLGA6L20	NM_001282503
107	66	hsa-miR-3130-5p	116443	GRIN3A	NM_133445
108	66	hsa-miR-3130-5p	79691	QTRTD1	NM_024638
109	66	hsa-miR-3130-5p	255967	PAN3	NM_175854
110	66	hsa-miR-3130-5p	4188	MDFI	NM_005586
111	66	hsa-miR-3130-5p	54848	ARHGEF38	NM_001242729
112	66	hsa-miR-3130-5p	112885	PHF21B	NM_001284296
113	66	hsa-miR-3130-5p	25992	SNED1	NM_001080437
114	66	hsa-miR-3130-5p	101929748	LOC101929748	XM_005252330
115	65	hsa-miR-3130-5p	25769	SLC24A2	NM_020344
116	65	hsa-miR-3130-5p	26091	HERC4	NM_001278187
117	65	hsa-miR-3130-5p	55751	TMEM184C	NM_018241
118	64	hsa-miR-3130-5p	635	BHMT	NM_001713
119	63	hsa-miR-3130-5p	55553	SOX6	NM_001145819
120	63	hsa-miR-3130-5p	23509	POFUT1	NM_015352
121	63	hsa-miR-3130-5p	55729	ATF7IP	NM_001286515
122	63	hsa-miR-3130-5p	79944	L2HGDH	NM_024884
123	63	hsa-miR-3130-5p	79727	LIN28A	NM_024674
124	63	hsa-miR-3130-5p	144165	PRICKLE1	NM_001144883
125	62	hsa-miR-3130-5p	56675	NRIP3	NM_020645

126	62	hsa-miR-3130-5p	10217	CTDSPL	NM_001008392
127	62	hsa-miR-3130-5p	7248	TSC1	NM_001162426
128	62	hsa-miR-3130-5p	64399	HHIP	NM_022475
129	62	hsa-miR-3130-5p	57690	TNRC6C	NM_001142640
130	61	hsa-miR-3130-5p	63934	ZNF667	NM_022103
131	61	hsa-miR-3130-5p	84466	MEGF10	NM_001256545
132	61	hsa-miR-3130-5p	84522	JAGN1	NM_032492
133	61	hsa-miR-3130-5p	78990	OTUB2	NM_023112
134	61	hsa-miR-3130-5p	7068	THRB	NM_000461
135	61	hsa-miR-3130-5p	10521	DDX17	NM_001098504
136	60	hsa-miR-3130-5p	26115	TANC2	NM_025185
137	60	hsa-miR-3130-5p	3551	IKBKB	NM_001242778
138	60	hsa-miR-3130-5p	200424	TET3	NM_001287491

Supplementary Table 3: list B of 59 up-regulated genes in the cases of PTSD&Dep versus controls in the genome-wide differential gene expression analysis in the GTP Discovery Sample

SYMBOL	p-value	Adjusted p-value
MRPS23	7.92486E-07	0.010340357
PDCD5	1.8437E-05	0.040595188
ERH	3.04734E-05	0.051649423
LRRC26	3.33504E-05	0.051649423
NOLA1	5.53596E-05	0.051649423
MRPS21	6.846E-05	0.051649423
SOD1	6.99802E-05	0.051649423
MRPS33	7.21617E-05	0.051649423
SHFM1	8.7101E-05	0.051649423
MT1X	8.72991E-05	0.051649423
RPLP2	8.89429E-05	0.051649423
ZNHIT3	9.13557E-05	0.051649423
UQCRH	0.000109654	0.057230858
C11orf1	0.000140607	0.062871718
GTF2H5	0.000153176	0.062871718
ZNF428	0.000154096	0.062871718
NDUFS5	0.000154294	0.062871718
RPL39	0.00015901	0.062871718
MGST3	0.000169051	0.063022163
RPS27L	0.000237385	0.069378545
PEBP1	0.000253017	0.069378545
MIF	0.000260171	0.069378545
RPL14	0.000291056	0.073032746
LSMD1	0.000304697	0.073623938
C14orf156	0.000333695	0.076363611
SEC61G	0.000348406	0.076363611
GTF3C6	0.00036031	0.076923573
PSMD14	0.000375045	0.076923573
RPS24	0.000387699	0.077078404
RPL32	0.000389882	0.077078404
CETN3	0.000403223	0.078293893
RPL26	0.000414031	0.078293893
C12orf62	0.000422252	0.078707828
RPL31	0.000516308	0.087772112
TRMT112	0.000524861	0.087772112
RPL41	0.000543672	0.087772112
NDUFA4	0.000545275	0.087772112
RPL37A	0.000560956	0.088184919
BOLA3	0.000570763	0.088658588

HAX1	0.000605024	0.088881323
RPL21	0.000621728	0.089146195
SHFM1	0.000636678	0.089518272
MRPL43	0.000644905	0.089518272
PHPT1	0.000667939	0.091739655
RPAIN	0.000697309	0.091911433
C19orf70	0.00070187	0.091911433
CKS2	0.000726862	0.091911433
NDUFA3	0.000738121	0.091911433
HAX1	0.000758281	0.091911433
TOMM7	0.000766703	0.091911433
MRPL35	0.000779956	0.091911433
RPL36	0.000791672	0.091911433
PFDN5	0.000813296	0.092462082
PRICKLE4	0.000814925	0.092462082
C19orf53	0.000863187	0.094731596
MRPS18C	0.000942448	0.098530398
SNRPF	0.000944286	0.098530398
RAP2A	0.00095858	0.098530398
UBE2T	0.000959025	0.098530398
